# Progress in Diseases of the Heart

**Published:** 1901-02-23

**Authors:** 


### PROGRESS IN DISEASES OF THE HEART.

(*Continued from page* 352.)

**Drugs.**—Sir T. Lauder Brunton[16] gives a report on the "Physiological and therapeutic action of digitalis and its active principles." He holds that digitalis acts directly in contracting the arterioles, and also stimulates the vasomotor centre in the medulla oblongata; it also increases the action of the heart. He cites the experiments of stimulating the vagus in a mammal—the heart's action is arrested and as a consequence the blood pressure falls rapidly; if, however, digitalis be previously administered, the blood pressure falls much more slowly. This must be due to the contraction of the arterioles. But it is uncertain whether the action is entirely upon the muscular fibres or upon the local nervous mechanism.

The diuretic effect is produced by its action in raising the tension of the renal arterioles. If the effect of the drug is strong, complete arrest may be a temporary result, as if the artery had been tied. As the blood pressure falls diuresis follows, and albumen is found in the urine.

He states that discord in the results of observers is probably due to differences in the chemical composition of the drug.

He points out that in a dilated heart the drug is useful in not only increasing the force of the beat and slowing the action, but also in lessening the ventricular dilatation during diastole and thus diminishing the amount of blood which can regurgitate through the relatively incompetent valves. It also, by contracting the arterioles, prevents the escape of the blood through the further end of the aorta, which renders the blood pressure in the aorta steady, less jerking, and more nearly normal. He advises with the use of the drug in these cases, massage and rest in bed.

In cases of fatty heart and very high tension digitalis may be harmful, by increasing the difficulty to a heart unable to respond to a further call on its energy. In these cases the tension must be lowered by the administration of nitrates, &c. The action of digitalis is rendered more efficient if mercury be added to the dose.

Floersheim[17] reports upon the use of extract of suprarenal capsule on the heart. There is no action upon the normal heart, but in cases of mitral regurgitation a rapid beneficial result occurs; the pulse grows fuller, stronger, and more regular, and the contractions of the heart more rhythmical.

**Interesting Cases.**—Professor Drasche[18] had the opportunity of watching a patient who was suffering from varicose veins of the lower extremities suddenly become half suffocated and cyanosed with loss of consciousness and convulsions. He examined the heart immediately and found that with an irregular, rapid pulse (150) there was a loud purring thrill over the levels of the second and third spaces, and at the same time there was a rough and prolonged systolic murmur. These two phenomena disappeared suddenly and the sounds of the heart were heard, though feebly. The patient lived for fifteen hours, when she died in a fresh attack of dyspnœa.

Post-mortem revealed the diagnosis of embolism of the pulmonary artery to be correct. The artery was filled with a thrombus, which reached the two main branches. There was extensive thrombosis in the veins of the lower extremities.

The first attack of dyspnœa had undoubtedly been caused by the passage of the clot through the auriculo-ventricular valve; and yet the patient lived for fifteen hours after this occurrence.

Dickinson and Fenton[19] report a case of complete coarctation of the arch of the aorta.

The symptoms were mainly a tumultuous and forcible action of the heart, and engorgement of the lungs. A faint systolic murmur was heard over the whole pericardium, and a diastolic murmur in the aortic area. The heart was dilated.

At the necropsy the arch of the aorta was found at first dilated and then narrowed as far as the junction of the ductus arteriosus, where there was a constricting ring completely obliterating the lumen. There were only two aortic valves, anterior and posterior, the coronary arteries arising behind the anterior cusp.

Everett[20] reports an interesting case of displacement of the heart.

The patient, a woman aged 60, presented while in fairly good health an impulse visible and palpable below the angle of the right scapula. On death—occurring from bronchitis—the necropsy revealed the left side occupied by the left lung, the lobes being arranged anteriorly ("upper") and posteriorly ("lower"). The anterior lobe extended across to the right side of the thorax, and being adherent to the costal pleura in the right mid-axilary line. The apex of the left side was occupied by both anterior and posterior lobes.

The pericardium occupied the right lower part of the thorax and rested on the diaphragm, and was altered in shape. The left side of the organ was forwards and a little to the left and the right backwards and a little to the right. The right ventricle therefore produced the impulse in the abnormal position.

Ewart[21] gives a case of fatal malignant endocarditis and right embolic hemiplegia, apparently due to a septic condition of the mouth. Antistreptococcic serum was used without avail.

[16] Lancet, Aug. 13. [17] Med. Rec., Oct. 13. [18] La Semaine Médicale, Oct. 17. [19] Lancet, Oct. 27. [20] Brit. Med. Jour., Sept. 8. [21] Brit. Med. Jour., Sept. 29.

